# Two Cases of Influenza B Virus-Related Fatal Fulminant Pneumonia Complicated With *Staphylococcus aureus* Infection in China Diagnosed Using Next-Generation Sequencing (2018)

**DOI:** 10.3389/fpubh.2020.00121

**Published:** 2020-04-15

**Authors:** Bing Bai, Hongyan Wang, Meng Li, Xiaoyu Ma, Jinxin Zheng, Qiwen Deng, Zhijian Yu

**Affiliations:** ^1^Shenzhen Key Laboratory for Endogenous Infection, Department of Infectious Diseases, Shenzhen Nanshan People's Hospital and the 6th Affiliated Hospital of Shenzhen University Health Science Center, Shenzhen, China; ^2^BGI Education Center, BGI Genomics and BGI-Shenzhen, University of Chinese Academy of Sciences, Shenzhen, China

**Keywords:** influenza B virus, *Staphylococcus aureus*, fatal pneumonia, sepsis, next-generation sequencing

## Abstract

Two rare cases of Chinese female patients with influenza B virus infection complicated with both fulminant pneumonia and septicemia caused by Panton–Valentine leukocidin(PVL) positive methicillin-sensitive *Staphylococcus aureus* (MASS) were reported for the first time in China through next-generation sequencing (NGS). An increasing body of evidence indicates that co-infection with influenza B virus and bacterial pneumonia is often fatal. Rapid and precise identification of the co-infection bacteria can guide the selection of treatment for patients with influenza virus infection in the clinical setting. In this study, next-generation sequencing (NGS) was applied for the rapid diagnosis of these two cases. Despite the unfavorable survival outcome of these patients, the application of next-generation sequencing showed promise as a diagnostic tool for the rapid diagnosis of unknown pathogens in patients with bacterial pneumonia and sepsis. This method can guide the administration of medications in such patients.

## Introduction

The influenza virus poses a health threat to human health worldwide and often predisposes individuals to person-to-person transmission (1). Co-infection with influenza virus and bacteria, most frequently *Streptococcus pneumonia* and *Staphylococcus aureus* (*S. aureus*), often worsen disease progression and prognosis (2–4). In recent years, co-infections with the influenza virus and *S. aureus* pneumonia have been increasingly reported worldwide (e.g., USA, Australia, Thailand, Hong Kong, and Taiwan) (5). Previous studies often reported that cases of influenza A or B virus infection were complicated with simple fulminant pneumonia caused by *S. aureus*. However, the clinical characteristics of influenza virus infection complicated with both fulminant pneumonia and septicemia caused by *S. aureus* were rarely mentioned and described (6–8). In this report we describe two cases of fatal sepsis and pneumonia caused by PVL-MSSA, which was following influenza B infection in the immunocompetent patients. To our knowledge, they are the first two cases reported in China in which co-incident infection with both influenza B and PVL-positive MSSA was laboratory confirmed. The bacterium was identified using next-generation sequencing (NGS) as a diagnostic tool.

## Case Report

### Case 1

On 13 January 2018, a previously healthy 64-year-old female with fever (38.5°C) was hospitalized in Shenzhen Nanshan People's Hospital (Shenzhen, China). The influenza-like symptoms (fever and cough) were initiated 5 days prior to admission. However, she was admitted to the hospital only after she experienced dyspnea. Following admission, her dyspnea was rapidly exacerbated, and endotracheal intubation was immediately applied in the intensive care unit. The results of the blood routine tests are shown in Table S1. The tracheal secretion obtained through bronchoscopy was analyzed for the detection of the influenza A or B virus using reverse transcription-polymerase chain reaction (reverse transcription-PCR). Rapid development of invasive shadows in the lung tissue was demonstrated by computed tomography and lung X-ray examination (Figure S1), indicating the rapid progression of fulminant pneumonia. The rescue process for this patient is shown in Figure S2. Antimicrobial treatment (i.e., ceftriaxone and linezolid) combined with antiviral therapy (i.e., oseltamivir and peramivir) were administered to the patient shortly after admission. Moreover, bronchial secretion and a blood sample were collected immediately for the detection of the pathogen using NGS at BGI-Shenzhen as previously reported; commonly-used bacterial culture methods in the microbiological laboratory were applied (9). After 5 h, the influenza B virus was identified in the tracheal secretion. Subsequently, methylprednisolone (80 mg/day) was administered. Furthermore, both continuous renal replacement therapy and extracorporeal membrane oxygenation (ECMO) were used as life support 14 h after admission. Unfortunately, the aggravating symptoms did not improve and the patient expired 20 h after admission. In addition, the family medical history of the patient revealed that two children in her family suffered from influenza-like symptoms for a week prior to the onset of her symptoms, possibly indicating the source of the influenza B virus infection in this patient. Four h after death (i.e., 24 h after admission), *S. aureus* was identified in the blood sample of the patient through both blood culture and the NGS approach. In contrast, *S. aureus* in the tracheal secretion was detected only through NGS. Further NGS analysis revealed that the *S. aureus* carried the Panton–Valentine leukocidin (PVL) genes (the virulence factors of *S. aureus* identified via NGS are shown in Table S2). The NGS identified 808 sequence reads (3.47834%) uniquely corresponding to the *S. aureus* genome; these reads covered a high percentage of the genome (shown in Figure S3). Therefore, this patient was diagnosed with co-infection with the influenza B virus and both pneumonia and sepsis caused by *S. aureus*.

### Case 2

On 4 February 2018, a 39-year-old female without any underlying diseases was admitted to the hospital due to fever and dyspnea lasting 2 days and 5 h, respectively. This patient experienced the influenza-like symptoms 2 days prior to hospitalization, and requested an ambulance due to the gradually exacerbating dyspnea 5 h prior to admission. The results of the laboratory tests are shown in Table S1. Multiple invasive development in the lung tissue was demonstrated by computed tomography and X-ray examination (shown in Figure S4). After admission, endotracheal intubation was immediately applied as the rescue approach, and antimicrobial treatment (i.e., moxifloxacin) combined with antiviral therapy (i.e., oseltamivir) were administered. The detection of the influenza B virus in the tracheal secretion was performed through reverse transcription-PCR. Moreover, bronchial secretion and a blood sample were collected immediately for pathogen detection using both NGS at BGI-Shenzhen and methods of bacterial culture commonly used in the clinical microbiological laboratory. After 4 h, the influenza B virus was identified in the tracheal secretion through reverse transcription-PCR. Subsequently, the patient received an injection with dexamethasone and ECMO was used as life support 8 h after admission. Unfortunately, the patient expired 11 h after admission. The details of the treatment process are shown in Figure S5. Twenty hours after admission, NGS at BGI-Shenzhen identified *S. aureus* in both the tracheal secretion and blood sample of this patient. Moreover, NGS identified 2,037 sequence reads (8.40934%) uniquely corresponding to the *S. aureus* genome; the identified *S. aureus* was a positive carrier of the PVL genes (Figure S6) (the virulence factors of *S. aureus* identified via NGS are shown in Table S2). The PVL genes were also amplified through PCR using the blood sample of this patient as previously described (3). In contrast, the commonly used bacterial culture methods were negative for the blood sample and tracheal secretion. Collectively, these results supported that the influenza virus B infection in this patient was complicated with fulminant pneumonia and sepsis caused by *S. aureus* infection.

## Discussion

Co-infection with the influenza B virus and bacterial pneumonia has been reported worldwide (3, 4). But to our knowledge, this is the first report of fatal fulminant pneumonia and septicemia in China in which co-incident infection with both influenza B and PVL-positive MSSA was laboratory confirmed. Several reports indicated that influenza virus infection results in epithelial cell injury and facilitates the occurrence of secondary bacterial infection through multiple mechanisms of immunological injury (3, 6–9). The hypothesis of immunological injury caused by the influenza virus B in the host includes decreased bacterial phagocytosis among alveolar macrophages, downregulated expression of toll-like receptors, and functional inhibition of neutrophil recruitment by type I interferon initiated by the viral infection (10). Moreover, hyperactive immune cell (T cells)-mediated cell immunity may aggravate the acute lung injury caused by influenza B virus infection and facilitate the occurrence of the secondary bacterial invasion (3, 10, 11).

An increasing body of evidence has shown that *Streptococcus pneumoniae* is the major pathogen involved in co-infections with the influenza virus. Co-infection with the influenza virus and *Streptococcus pneumoniae* may be responsible for the high morbidity and mortality observed in the 1918–1919 influenza pandemic (2). In recent years, *S. aureus* is gradually emerging as another important pathogen involved in co-infection with the influenza virus, often leading to fatal pneumonia as shown in the present two cases. A report from the United States of America indicated that co-infection with the influenza virus A and PVL-positive *S. aureus* may be responsible for the 56 of the 62 cases (90%) of histologically confirmed fatal necrotizing pneumonia (7).

Previous reports often focused on the characteristics of disease progression in patients co-infected with the influenza virus and *S. aureus* pneumonia. In the present report, two cases of influenza B Virus infection complicated with fatal pneumonia and sepsis caused by PVL-positive *S. aureus* were described. Moreover, we conducted a literature review and found that two other cases of this co-infection have been documented in the USA between 2008 and 2014 (Table 1). This suggests that the two patients remained healthy without underlying diseases prior to the onset of the influenza B virus infection, and manifested with the rapid aggravation of respiratory failure, leading to the rapid application of ECMO. During hospitalization, *S. aureus* infection in both the tracheal secretions and blood samples of these two patients was determined through commonly used bacterial culture methods. To the best of our knowledge, this is the first report of fatal fulminant pneumonia caused by influenza B complicated with pneumonia and septicemia caused by PVL-positive methicillin-sensitive *S. aureus* infection in China. Notably, co-infection in these two patients developed rapidly. Septicemia caused by *S. aureus* may be an important factor contributing to the worsening of prognosis in these two cases. Therefore, we hypothesized that the co-infection with the influenza virus B and *S. aureus* in the lung and bloodstream contributed to the aggravation of the fatal pneumonia and disease progression in these two cases (1–4).

**Table 1:** Case reports of fatal fulminant pneumonia caused by PVL-positive community-associated *S. aureus* and influenza B co-infection.

**Age**	**Sex**	**Location**	**Year**	**Drug treatment**	**Clinical diagnosis**	**Etiological diagnosis**	**Days from the onset of symptoms to death**	**References**
38	Female	New York Jersey, USA	2012	Oseltamivir, ceftriaxone, vancomycin, azithromycin, linezolid	Pneumonia; ARDS	1. Influenza B virus (culture of the nasal swab); 2. MRSA (culture of the nares and blood)	14 days	(4)
30	Female	Tampa, Florida, USA	2007	Ceftriaxone, moxifloxacin, gentamycin, ciprofloxacin, piperacillin, tazobactam	Necrotizing pneumonia; septic shock	1. Influenza B virus (RT-PCR from blood and lung); 2. MSSA (culture of blood and lung tissues)	6 days	(16)
64	Female	Shenzhen Guangdong, China	2018	Ceftriaxone, linezolid, oseltamivir, peramivir	Fulminant pneumonia; sepsis	1. Influenza B virus (RT-PCR from tracheal secretion); 2. MSSA (NGS from tracheal section and blood sample)	5 days	In this study
39	Female	Shenzhen, Guangdong, China	2018	Oseltamivir, moxifloxacin, vancomycin, metropenem	CAP; sepsis	1. Influenza B virus (RT-PCR from tracheal secretion); 2. MSSA (NGS from tracheal section and blood sample)	3 days	In this study

*PVL, Panton–Valentine leukocidin; ARDS, acute respiratory distress syndrome; CAP, community-acquired pneumonia; MRSA, methicillin-resistant Staphylococcus aureus; RT-PCR, reverse transcription polymerase chain reaction; MSSA, methicillin-sensitive Staphylococcus aureus; NGS, next-generation sequencing*.

NGS was introduced for routine diagnostics in 2014, and the majority of indications are outbreak investigation and genotyping of highly resistant micro-organisms. Pathogenic microorganism DNA/RNA high-quality sequencing data were classified by simultaneously aligning to four Microbial Genome Databases, consisting of viruses, bacteria, fungi, and parasites. The classification reference databases were downloaded from NCBI (ftp://ftp.ncbi.nlm.nih.gov/genomes/). RefSeq contains 4,061 whole genome sequence of viral taxa, 2,473 bacterial genomes or scaffolds, 199 fungi related to human infection, and 135 parasites associated with human diseases. NGS played a critical role in the accurate diagnosis of *S. aureus* infection in these two cases. *S. aureus* infection in the blood sample was identified through bacterial culture only in Case 1. In contrast, NGS provided rapid detection of *S. aureus* in both the blood samples and tracheal secretions of these two patients. Furthermore, NGS offers microbial detection in clinical specimens within a 24–30 h period. Therefore, this approach facilitates the rapid identification of unknown pathogens in clinical specimens to guide the selection of appropriate treatment (12–14). In this study, the virulence factors of *S. aureus* were identified through NGS in the blood samples and bronchial secretions, demonstrating that both patients were infected with PVL-positive *S. aureus*. Multiple reports indicated the critical pathological role of PVL in *S. aureus*-caused fatal pneumonia complicated with influenza virus infection (3, 10, 11, 15). Moreover, previous case reports indicated that necrotizing pneumonia caused by PVL-positive *S. aureus* is often associated with poor prognosis and a specific mortality rate of ~75% (3). Notably, some studies indicated the participation of toxic shock syndrome toxin-1 and enterotoxin B in fatal pneumonia caused by co-infection with the influenza B virus and *S. aureus* (8). Therefore, the influence and mechanism of the virulence factors of *S. aureus* on the clinical outcome of co-infection with the influenza virus and *S. aureus* warrant further assessment.

Recent reports have suggested that therapy with corticosteroids exerts beneficial effects in the early stage of severe community-acquired pneumonia; however, its use for the treatment of severe pneumonia caused by influenza virus infection remains controversial (16). The early use of antimicrobial agents (e.g., antibiotics or antiviral agents) combined with systemic immune modulators (e.g., corticosteroids and/or high-dose intravenous immunoglobulin) are effective measures for preventing the rapid progression of viral pneumonia and reducing the risk of subsequent bacterial pneumonia (3, 8). Treatment with antiviral agents is crucial for the rapid resolution of pulmonary lesions in pneumonia caused by the influenza virus. Antiviral agents, such as oseltamivir and peramivir, should be used as early as possible during the initial stage of respiratory distress to improve the prognosis of the patients. However, antiviral therapy with both oseltamivir and peramivir is seldom administered. In this study, two patients received treatment with antiviral agents very early after admission and rapid life support measures (i.e., ECMO). However, it was not possible to block the rapid disease progression, indicating the poor prognosis linked to co-infection with the influenza virus and *S. aureus-*caused pneumonia and sepsis.

Conclusively, co-infection with the influenza B virus and pneumonia and sepsis caused by *S. aureus* can be fatal. Physicians should pay special attention to the worsening prognosis associated with this co-infection. In addition, NGS showed its critical role in the accurate diagnosis of the pathogen in infected patients with unknown etiology. As a complementary method of bacterial culture, NGS can provide rapid detection of pathogens in both blood samples and tracheal secretions obtained from infected patients. Furthermore, this method can provide, at least partly, information regarding the virulence factors of pathogens.

## Data Availability Statement

The RNA sequence of virulence factors assembled using the next-generation sequencing data are listed in Supplementary Material.

## Ethics Statement

The institutional ethical committee of Shenzhen Nanshan People's Hospital approved this case report. Written informed consent was obtained from members of the patient's family for the publication of this case report.

## Author Contributions

BB, HW, XM, and ZY collected the medical data of the patients and drafted the manuscript. ML and XM participated in bibliographic research. ML, XM, and JZ were involved in the gene sequence analysis and susceptibility test. All authors critically reviewed the manuscript. QD and ZY drafted and revised the article. All authors read and approved the final version of the article.

### Conflict of Interest

The authors declare that the research was conducted in the absence of any commercial or financial relationships that could be construed as a potential conflict of interest.

## Abbreviations

*S. aureus*: *Staphylococcus aureus*
NGS: next-generation sequencing
ECMO: extracorporeal membrane oxygenation
PCR: polymerase chain reaction
PVL: Panton–Valentine leukocidin.
