# Supplementary material for: Two Cases of Influenza B Virus-Related Fatal Fulminant Pneumonia Complicated With Staphylococcus aureus Infection in China Diagnosed Using Next-Generation Sequencing (2018)
Source: Front Public Health. 2020 Apr 15;8:121. doi: 10.3389/fpubh.2020.00121 (PMC7175880; doi:10.3389/fpubh.2020.00121)
Supplement: Supplementary file 1 [file Data_Sheet_1.doc]

**Case1**

**Table S1 the dynamics results of laboratory tests during case1 disease progression**

| Date | 13/1/2018 | 13/1/2018 | 13/1/2018 | 14/1/2018 | 14/1/2018 | 14/1/2018 | 14/1/2018 | 14/1/2018 |
| --- | --- | --- | --- | --- | --- | --- | --- | --- |
| Time | 18:40:00 | 23:22:00 | 23:28:00 | 3:52:00 | 5:48:00 | 8:45:00 | 11:47:00 | 15:57:00 |
| total white blood cells count(×10^9^) | 5.6 | 0.7 | 0.7 | 1.6 | 1.6 | 1.3 | 1.5 | 0.7 |
| Rate of Neutrophils | 71.3 | 64.2 | 64.2 | 32.9 | 33 | 31.8 | 40.7 | 45.8 |
| Rate of lymphocytes | 21.4 | 34.3 | 34.3 | 63.3 | 59.6 | 60.6 | 53.8 | 48.6 |
| rate of monocytes | 7.1 | 1.5 | 1.5 | 3.2 | 6.2 | 6.8 | 3.4 | 4.2 |
| rate of eosinophils | 0 | 0 | 0 | 0.6 | 0.6 | 0.8 | 1.4 | 1.4 |
| tate of basophils | 0.2 | 0 | 0 | 0 | 0.6 | 0 | 0.7 | 0 |
| Neutrophils count(×10^9^) | 4 | 0.43 | 0.43 | 0.52 | 0.53 | 0.42 | 0.59 | 0.33 |
| lymphocytes count(×10^9^) | 1.2 | 0.23 | 0.23 | 1 | 0.96 | 0.8 | 0.78 | 0.35 |
| monocytes count(×109) | 0.4 | 0.01 | 0.01 | 0.05 | 0.1 | 0.09 | 0.05 | 0.03 |
| eosinophils count (×10^9^) | 0 | 0 | 0 | 0.01 | 0.01 | 0.01 | 0.02 | 0.01 |
| basophils count(×10^9^) | 0.01 | 0 | 0 | 0 | 0.01 | 0 | 0.01 | 0 |
| Erythrocytes count(×10^12^) | 4.35 | 4.78 | 4.78 | 5.21 | 2.71 | 1.77 | 2.18 | 2.24 |
| Hemoglobin concentration | 132 | 144 | 144 | 156 | 81 | 53 | 62 | 66 |
| C-reactive protein(CRP,mg/L) | - | - | 10.7 | 20.5 | - | - | 4.5 | - |
| procalcitonin(PCT,mg/L) | 6.3 | - | - | - | - | - | - | - |


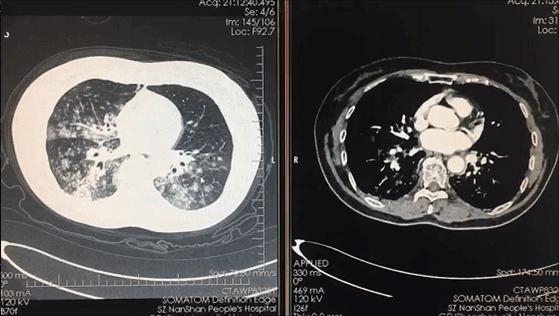


A

B


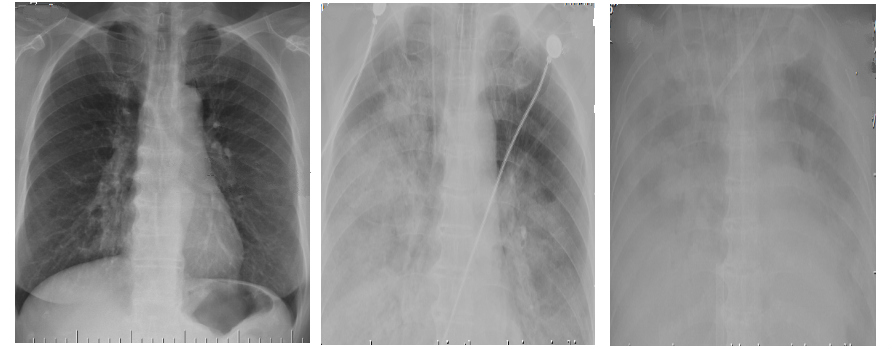


C

D

E

Fig S1 The invasive inflammation shadows in the lung by the CT scan and X-ray examinations of case1 during the disease progression. The CT scan after 2h of her admission was shown in 1A and 1B, indicating mass structure messy and irregular huge mass shadow. The X-ray examinations at 1h before her hospitalization and 10h and 19h after her hospitalization were shown in 1C,1D and 1E respectively, indicating rapid development of the pulmonary exudative lesions.

**dead**

+endotracheal intubation+ceftriaxone+linezolid

oseltamivir 150mg Bid+ peramivir 600mg+ moxifloxacin 0.4g Qd

Dexamethasone

**5mg**

Methylprednisolone **80mg**

**Sudden cardiac arrest for 3 times**

**CDC: Influenza**

**B virus**

+CRRT+ECMO

Time after her admission (h)

0

12

4

16

8

20

Fig S2 The treatment details of Case1 after her admission

**A**

**B**

**C**

Fig S3 Diagnosis of *S aureus* infection of case1 using the NGS methods in the blood samples of the case 1. A. Mapping of S aureus reads on the genome. B. Reads distribution of total DNA in the blood sample. C. Reads distribution of microbes and unkown reads in the absence of human host reads.

**Table S2 The virulence factors analysis of *S. aureus* by NGS from the blood samples of case1 exhibiting sequence coverage≥10%**

| Virulence factors of case1-originated S aureus from case1 | | | |
| --- | --- | --- | --- |
| ID▲ | Virulence Factor△ | Average depth● | Coverage（%）★ |
| VFG004543(gi:87161717) | icaA | 0.11 | 10.5 |
| VFG001311(gb|NP_644953) | cap8O | 0.2 | 10.86 |
| VFG004889(gi:57651313) | set38 | 0.11 | 11.12 |
| VFG004804(gi:87160716) | hlgB | 0.11 | 11.16 |
| VFG001798(gb|YP_186826) | hlb | 0.11 | 11.39 |
| VFG005018(gi:82749866) | capE | 0.13 | 12.94 |
| VFG044256(gi:151220274) | sbnC | 0.13 | 13.06 |
| VFG043587(gi:57652054) | sasC | 0.15 | 13.28 |
| VFG043452(gi:57651346) | SACOL0507 | 0.13 | 13.35 |
| VFG004773(gb|NP_647407) | lip | 0.14 | 13.84 |
| VFG001318(gb|YP_001332075) | isdA | 0.14 | 13.88 |
| VFG004842(gi:87160380) | hly/hla | 0.14 | 14.39 |
| VFG044258(gi:151220276) | sbnE | 0.15 | 14.57 |
| VFG004877(gi:82750780) | eta | 0.15 | 14.78 |
| VFG044273(gi:151222290) | htsA | 0.15 | 14.85 |
| VFG003449(gi:87160783) | cap5C | 0.16 | 15.58 |
| VFG005102(gi:49482404) | capM | 0.16 | 15.62 |
| VFG002165(gb|NP_815739) | efaA | 0.16 | 15.66 |
| VFG002420(gb|NP_644838) | adsA | 0.16 | 16.05 |
| VFG044257(gi:151220275) | sbnD | 0.25 | 17.2 |
| VFG044262(gi:151220280) | sbnI | 0.17 | 17.28 |
| VFG001319(gb|YP_001332076) | isdC | 0.17 | 17.42 |
| VFG004463(gi:49482393) | capB | 0.17 | 17.49 |
| VFG004733(gi:82750500) | nuc | 0.18 | 18.08 |
| VFG001298(gb|NP_644940) | cap8B | 0.19 | 18.95 |
| VFG001312(gb|NP_644954) | cap8P | 0.19 | 19.13 |
| VFG001274(gb|NP_647160) | hlgC | 0.22 | 22.07 |
| VFG004458(gi:87161167) | cap5B | 0.4 | 22.3 |
| VFG044251(gi:151220269) | sirC | 0.22 | 22.34 |
| VFG044252(gi:151220270) | sirB | 0.37 | 27.04 |
| VFG044261(gi:151220279) | sbnH | 0.33 | 32.95 |
| VFG044260(gi:151220278) | sbnG | 0.62 | 33.12 |
| VFG044259(gi:151220277) | sbnF | 0.54 | 36.38 |
| VFG044255(gi:151220273) | sbnB | 0.47 | 37.03 |
| VFG001276(gb|NP_646195) | lukF-PV | 0.49 | 37.36 |
| VFG001277(gb|NP_646196) | lukS-PV | 0.69 | 47.87 |
| VFG044254(gi:151220272) | sbnA | 0.51 | 50.82 |

▲represent the genebank number; △, the common names of the virulence factors; ●,the average depth of sequence;

★ the coverage of full-length sequence of virulence factors. The shadow colomn showed the *S. aureus* pvl gene tests of each patients.

**Case 2**

**Table S3 the dynamics results of laboratory tests during case2 disease progression.**

| Date | 4/2/2018 | 4/2/2018 | 4/2/2018 |
| --- | --- | --- | --- |
| Time | 8:26:00 | 12:43:00 | 16:30:00 |
| total white blood cells count(×10^9^) | 2.38 | 1.85 | 4.7 |
| Rate of Neutrophils | 75.2 | 72 | 37.3 |
| Rate of lymphocytes | 10.9 | 20.5 | 53.4 |
| rate of monocytes | 13.9 | 7 | 8.9 |
| rate of eosinophils | 0 | 0 | 0.2 |
| tate of basophils | 0 | 0.5 | 0.2 |
| Neutrophils count(×10^9^) | 1.79 | 1.33 | 1.75 |
| lymphocytes count(×10^9^) | 0.26 | 0.38 | 2.51 |
| monocytes count(×109) | 0.33 | 0.01 | 0.42 |
| eosinophils count (×10^9^) | 0 | 0 | 0.01 |
| basophils count(×10^9^) | 0 | 0.01 | 0.01 |
| Erythrocytes count(×10^12^) | 5.17 | 5.66 | 6.42 |
| Hemoglobin concentration | 144 | 158 | 178 |
| C-reactive protein(CRP,mg/L) | - | - | 94.4 |
| procalcitonin(PCT,mg/L) | - | 11.04 | - |


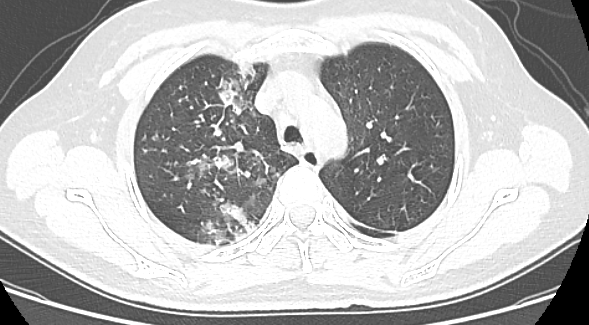

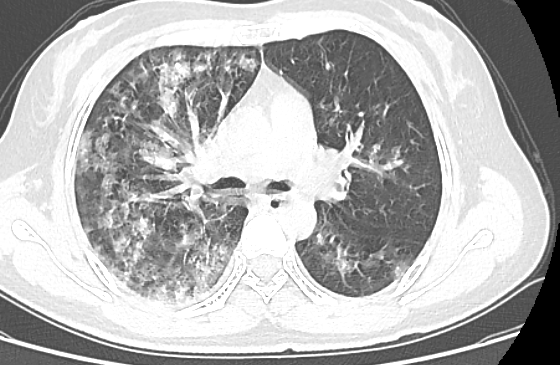

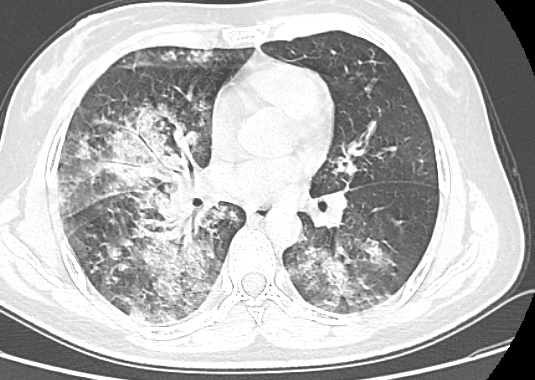

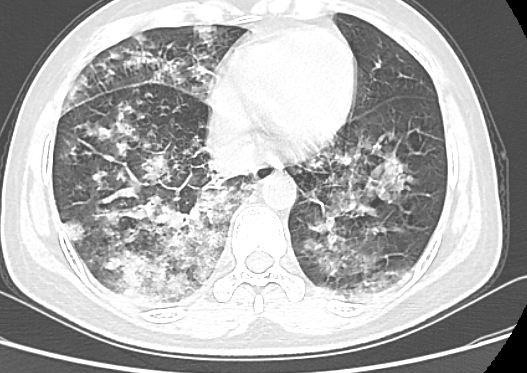


**A**

**B**

**C**

**D**


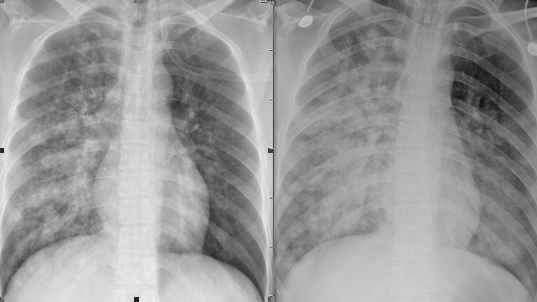


**E**

**F**

Fig S4 The invasive inflammation shadows in the lung by the CT scan and X-ray examination of case 2 during the disease progression. The CT scan of case 2 before ten minutes of her admission was shown in A, B, C and D, indicating scattered shadow of the patchy fuzzy image. The X-ray examinations of case 2 before 1h and after 4h of her admission were shown in E and F, indicating the fast development of the pulmonary exudative lesions.

**dead**

**+endotracheal intubation**

oseltamivir **150mg Bid+** moxifloxacin **0.4g Qd**

Dexamethasone

**5mg**

Methylprednisolone **120mg**

**CDC: Influenza**

**B virus**

**+ECMO+** peramivir **600mg**

Time after her admission (h)

0

6

2

8

4

11

**+Metropenem 1.0g Q8h**

**+Vancomycin 100IU Q8h**

Fig S5 The treatment details of Case2 after her admission

**A**

**B**

**C**

Fig S6 Diagnosis of S aureus infection using the NGS method in the blood samples of the case 2. A. mapping of S aureus reads on the genome.B. reads distribution of total DNA in the CSF sample. C. Reads distribution of microbes and unkown reads in the absence of human host reads.

**Table S4 The virulence factors analysis of *S. aureus* by NGS from the blood samples of case2 exhibiting sequence coverage≥10%**

| Virulence factors of case2-originated S aureus from case2 | | | |
| --- | --- | --- | --- |
| ID▲ | Virulence Factor△ | Average depth● | Coverage（%）★ |
| VFG002420(gb|NP_644838) | adsA | 0.45 | 34.9 |
| VFG004483(gi:82750663) | atl | 0.17 | 17.01 |
| VFG005085(gi:87162167) | cap5L | 0.12 | 12.2 |
| VFG001299(gb|NP_644941) | cap8C | 0.16 | 16.1 |
| VFG001303(gb|NP_644945) | cap8G | 0.1 | 10.41 |
| VFG001311(gb|NP_644953) | cap8O | 0.12 | 11.89 |
| VFG001312(gb|NP_644954) | cap8P | 0.38 | 38.08 |
| VFG003450(gi:82749864) | capC | 0.18 | 17.54 |
| VFG005018(gi:82749866) | capE | 0.11 | 11.38 |
| VFG005025(gi:49482397) | capF | 0.21 | 20.92 |
| VFG005040(gi:82749868) | capG | 0.24 | 24.02 |
| VFG005091(gi:49482403) | capL | 0.11 | 11.37 |
| VFG005122(gi:49482406) | capO | 0.13 | 12.68 |
| VFG005131(gi:49482407) | capP | 0.22 | 16.51 |
| VFG004656(gi:82749938) | coa | 0.33 | 23.21 |
| VFG004503(gi:82751025) | ebh | 0.17 | 15.04 |
| VFG004491(gi:87162306) | efb | 0.21 | 20.72 |
| VFG004494(gi:15926741) | efb | 0.25 | 25.35 |
| VFG043451(gi:57650133) | emp | 0.24 | 23.68 |
| VFG005576(gi:76788358) | eno | 0.11 | 10.71 |
| VFG002407(gb|NP_645076) | esaB | 0.55 | 54.96 |
| VFG018118(gi:151220433) | essA | 0.28 | 27.95 |
| VFG018141(gi:156978615) | essB | 0.12 | 11.77 |
| VFG002405(gb|NP_645073) | esxA | 0.48 | 47.78 |
| VFG004878(gi:88194875) | eta | 0.14 | 13.83 |
| VFG004839(gi:15924993) | hlb | 0.18 | 17.95 |
| VFG001273(gb|NP_647159) | hlgA | 0.16 | 15.93 |
| VFG004816(gi:15927998) | hlgC | 0.14 | 14.47 |
| VFG001274(gb|NP_647160) | hlgC | 0.15 | 14.89 |
| VFG001293(gb|NP_645861) | hly/hla | 0.15 | 15.02 |
| VFG044272(gi:151222289) | htsB | 0.85 | 49.37 |
| VFG001285(gb|NP_647403) | icaA | 0.21 | 20.76 |
| VFG004558(gi:49484860) | icaR | 0.26 | 26.07 |
| VFG001318(gb|YP_001332075) | isdA | 0.31 | 27.47 |
| VFG001319(gb|YP_001332076) | isdC | 0.2 | 20.35 |
| VFG044268(gi:151221257) | isdF | 0.14 | 13.53 |
| VFG001324(gb|YP_001332081) | isdG | 0.49 | 49.23 |
| VFG044443(gi:151221836) | isdH | 0.14 | 14.33 |
| VFG004773(gb|NP_647407) | lip | 0.25 | 18.88 |
| VFG006776(gi:16800002) | lplA1 | 0.13 | 12.86 |
| VFG001277(gb|NP_646196) | lukS-PV | 0.51 | 34.22 |
| VFG004732(gi:87161840) | nuc | 0.11 | 10.93 |
| VFG005353(gi:76787761) | plr/gapA | 0.13 | 12.97 |
| VFG043452(gi:57651346) | SACOL0507 | 0.69 | 59.86 |
| VFG043587(gi:57652054) | sasC | 0.16 | 14.19 |
| VFG043588(gi:57651004) | sasG | 0.18 | 11.74 |
| VFG002421(gb|NP_647158) | sbi | 0.21 | 19.8 |
| VFG044254(gi:151220272) | sbnA | 0.63 | 51.33 |
| VFG044255(gi:151220273) | sbnB | 0.39 | 33.17 |
| VFG044256(gi:151220274) | sbnC | 0.45 | 36.15 |
| VFG044257(gi:151220275) | sbnD | 0.74 | 52.55 |
| VFG044259(gi:151220277) | sbnF | 0.79 | 49.3 |
| VFG044260(gi:151220278) | sbnG | 0.3 | 19.51 |
| VFG044261(gi:151220279) | sbnH | 0.89 | 53.58 |
| VFG044262(gi:151220280) | sbnI | 0.48 | 47.77 |
| VFG001279(gb|NP_645333) | sdrC | 0.17 | 15.56 |
| VFG004939(gi:15926106) | set13 | 0.16 | 16.19 |
| VFG004910(gi:82750141) | set23 | 0.21 | 21.49 |
| VFG004904(gi:82750143) | set25 | 0.21 | 21.24 |
| VFG004899(gi:87160089) | set30 | 0.21 | 20.74 |
| VFG004897(gi:87161928) | set31 | 0.2 | 19.71 |
| VFG004894(gi:87160066) | set33 | 0.15 | 14.9 |
| VFG044275(gi:151222292) | sfaB | 0.22 | 21.57 |
| VFG044274(gi:151222291) | sfaC | 0.11 | 10.65 |
| VFG044277(gi:151222294) | sfaD | 0.38 | 30.97 |
| VFG044253(gi:151220271) | sirA | 0.73 | 52.42 |
| VFG044252(gi:151220270) | sirB | 0.6 | 51.36 |
| VFG044251(gi:151220269) | sirC | 0.88 | 57.82 |
| VFG001313(gb|NP_644899) | spa | 0.13 | 13.33 |
| VFG043589(gi:57652394) | sraP | 0.26 | 21.95 |
| VFG004778(gi:87160772) | sspA | 0.11 | 11.09 |
| VFG004782(gi:49485888) | sspA | 0.13 | 12.69 |
| VFG001295(gb|NP_645748) | sspB | 0.11 | 10.58 |
| VFG005549(gi:116516755) | tig/ropA | 0.12 | 12.24 |
| VFG001276(gb|NP_646195) | lukF-PV | 0.04 | 4.3 |

▲represent the genebank number; △, the common names of the virulence factors; ●,the average depth of sequence;

★ the coverage of full-length sequence of virulence factors. The shadow colomn showed the *S. aureus* pvl gene tests of each patients.
